# Pregnancy in the mature adult mouse does not alter the proportion of mammary epithelial stem/progenitor cells

**DOI:** 10.1186/bcr2245

**Published:** 2009-04-23

**Authors:** Kara L Britt, Howard Kendrick, Joseph L Regan, Gemma Molyneux, Fiona-Ann Magnay, Alan Ashworth, Matthew J Smalley

**Affiliations:** 1 Breakthrough Breast Cancer Research Centre, The Institute of Cancer Research, 237 Fulham Road, London SW3 6JB, UK; 2 Monash Institute of Medical Research, Monash Medical Centre, Clayton Road, Clayton 3168, Australia

## Abstract

**Introduction:**

In humans, an early full-term pregnancy reduces lifetime breast cancer risk by up to 50% whereas a later pregnancy (>35 years old) can increase lifetime risk. Several mechanisms have been suggested, including changes in levels of circulating hormones, changes in the way the breast responds to these hormones, changes in gene expression programmes which may alter susceptibility to transformation and changes to mammary stem cell numbers or behaviour. Previous studies have shown that the mammary tissue isolated from both virgin and parous mice has the ability to repopulate a cleared mammary fat pad in transplant experiments. Limited dilution transplant assays have demonstrated that early pregnancy (at 5 weeks of age) reduces stem/progenitor cell numbers in the mouse mammary epithelium by twofold. However, the effects on stem/progenitor cell numbers in the mammary epithelium of a pregnancy in older animals have not yet been tested.

**Methods:**

Mice were put through a full-term pregnancy at 9 weeks of age, when the mammary epithelium is mature. The total mammary epithelium was purified from parous 7-week post-lactation and age-matched virgin mice and analysed by flow cytometry and limiting dilution cleared fat pad transplants.

**Results:**

There were no significant differences in the proportions of different mammary epithelial cell populations or numbers of CD24^+/Low ^Sca-1^- ^CD49f^High ^cells (stem cell enriched basal mammary epithelial compartment). There was no significant difference in stem/progenitor cell frequency based on limiting dilution transplants between the parous and age-matched virgin epithelium.

**Conclusions:**

Although differences between parous and virgin mammary epithelium at later time points post lactation or following multiple pregnancies cannot be ruled out, there are no differences in stem/progenitor cell numbers between mammary epithelium isolated from parous animals which were mated at 9 weeks old and virgin animals. However, a recent report has suggested that animals that were mated at 5 weeks old have a twofold reduction in stem/progenitor cell numbers. This is of interest given the association between early, but not late, pregnancy and breast cancer risk reduction in humans. However, a mechanistic connection between stem cell numbers and breast cancer risk remains to be established.

## Introduction

It is well established that pregnancy has a profound effect on breast cancer risk [1] (reviewed in [2]). Breast cancer risk is significantly increased immediately after parturition and this elevated risk can last for a period of 5 to 10 years in humans [3,4]. Once this elevated risk period is past, however, breast cancer risk drops in women who have had an early first full-term pregnancy to below the levels of nulliparous women of a similar age. The eventual risk decrease is more profound the earlier the age at which the first full-term pregnancy occurs [1,3,5]; a woman who has a first full-term pregnancy under the age of 20 can reduce her lifetime risk of breast cancer by 50%, even allowing for the increased risk period immediately after parturition [1]. In contrast, first full-term births over the age of 35 result in an elevated overall lifetime breast cancer risk [5]. Interestingly, this lifetime protective effect is limited to oestrogen receptor-positive/progesterone receptor-positive (ER^+^PR^+^) breast cancers and is not seen for other breast tumour types [2]. Both parity and parity-like oestrogen treatment can also protect rodents against the development of hormone-dependent carcinogen-induced mammary tumours [6-8].

The mechanism or mechanisms behind these effects remain unclear, although several have been proposed and we have recently discussed these in detail [2]. In brief, four potential mechanisms have received most attention. First, changes in levels of circulating hormones such as oestradiol, prolactin and growth hormone may mediate the effects of pregnancy on breast cancer risk. Second, changes in the way the mammary epithelium senses hormones after pregnancy may alter its response to them. Third, changes in gene expression programmes in mammary cells after pregnancy may alter the susceptibility of the cells to transformation. Finally, the proliferative stimulus of pregnancy on stem cells and alveolar progenitors with pre-existing genetic lesions might account for the increase in breast cancer risk during and immediately after pregnancy. Conversely, a proportion of mammary stem/progenitor cells that do not carry mutations (presumably the majority) could be forced to permanently differentiate by the stimulus of pregnancy, resulting in a reduction in the numbers of a cell population at high risk of transformation [9] and therefore a reduction in the lifetime risk of breast cancer.

In the present study, we address the possibility that the alterations in the ER^+^PR^+ ^breast cancer risk profile caused by parity are a result of changes in mammary epithelial stem/progenitor activity following pregnancy. Epidemiological studies have shown that women who are prenatally exposed to restricted oestrogens (associated with preeclampsia) have a decreased risk of developing breast cancer, whilst those who are prenatally exposed to increased levels (diethylstilbestrol or oestrogen treatment) or who experience menarche (associated with high oestrogen levels) at a young age are at an increased risk (reviewed in [2]). This sensitivity to oestrogen occurs at stages when mammary stem cells are thought to be the most active in the developing breast (during prenatal development and during puberty) [7] and are undergoing both symmetric and asymmetric divisions to produce the stem cell pool and differentiated daughters required for the mature functional mammary gland. Furthermore, although the mammary epithelial stem cells of the adult gland are ERα-negative [10,11], they may still be responsive to oestrogen exposure. In ER^- ^spermatogonial and endometrial stem cells, oestrogen has been shown to affect stem cell proliferation [12,13] – and we have recently demonstrated that ER^+ ^differentiated mammary epithelial cells do not have an oestrogen-responsive gene signature as defined by other studies. Rather, many of the genes whose expression has previously been reported to be stimulated by oestrogen in breast cancer cell lines [14-16] are actually most strongly expressed in basal/myoepithelial cells [17]. The effects of oestrogen on adult stem cells may therefore be partly ER independent. Also clear, however, is that oestrogen affects ER^- ^mammary stem cells via paracrine interactions with ER^+ ^differentiated cells [17-21].

Until recently, only limited direct experimental evidence existed on whether mammary stem behaviour is altered by pregnancy. A previous study that assessed transplantation rates in virgin mice and parous regressed animals found no differences [22]. Whether the virgin and parous animals were age matched, or for how many weeks post weaning the parous animals had been allowed to involute, however, was unclear. Furthermore, the study used microdissected pieces of tissue, not limited dilution assays of known cell numbers, so the numbers of cells transplanted cannot be compared. While the current manuscript was being prepared, however, Siwko and colleagues reported a study in which pregnancy in young mice (mated at 5 weeks of age) resulted in a twofold reduction in the numbers of stem/progenitor cells [23].

The mammary epithelium is composed of two main cell types, the basal cells and the luminal cells. The basal cell layer is mainly composed of myoepithelial cells but also contains the mammary epithelial stem cell compartment in both the mouse and human breast [11,24-28]. The mammary epithelium is also thought to contain a number of progenitor cell types downstream of the stem cells, with good evidence existing in particular for a progenitor cell for the milk-producing alveoli of pregnancy in the luminal epithelial cell layer [28-32]. Evidence also exists from bromodeoxyuridine labelling strategies for a distinct lineage of progenitors for ER-expressing cells [33,34].

We have previously used a cell separation strategy based on expression of CD24 and Sca-1 to isolate and characterise primary mouse mammary cells from 10-week-old to 12-week-old virgin mice. We showed that the basal cell population has a CD24^+/Low ^Sca-1^- ^staining pattern, the luminal ER^- ^cells are CD24^+/High ^Sca-1^- ^and the luminal ER^+ ^cells are CD24^+/High ^Sca-1^+^. We also showed that the ER^+ ^cells of the mammary gland have little or no transplant activity in cleared fat pad transplant assays but that, consistent with other reports, stem cell activity is concentrated in the basal cell layer and can be enriched by isolation of CD24^+/Low ^cells that express high levels of α_6 _integrin (CD49f) [11,26,27]. No strategy yet exists for unequivocal isolation of pure basal stem cells, however, and some progenitor activity can be identified in the other populations [11]. We therefore employed our CD24 and Sca-1 sorting strategy to purify the total epithelial compartment from the mammary fat pads of parous 7-week post-lactation and age-matched virgin (AMV) mice, and analysed these total epithelial preparations by limited dilution transplantation for changes in stem/progenitor cell activity. We used mice that were mated at 9 weeks of age, when the mammary epithelium is essentially fully mature, as opposed to Siwko and colleagues who mated their animals during mid-puberty [23]. In contrast to Siwko and colleagues, we find that a pregnancy in mice with developmentally mature mammary tissue does not affect stem/progenitor cell numbers. Taken together, our results and those of Siwko and colleagues support a model in which early, but not late, pregnancy depletes mammary epithelial stem cells.

## Materials and methods

### Animals

**Figure 1:**
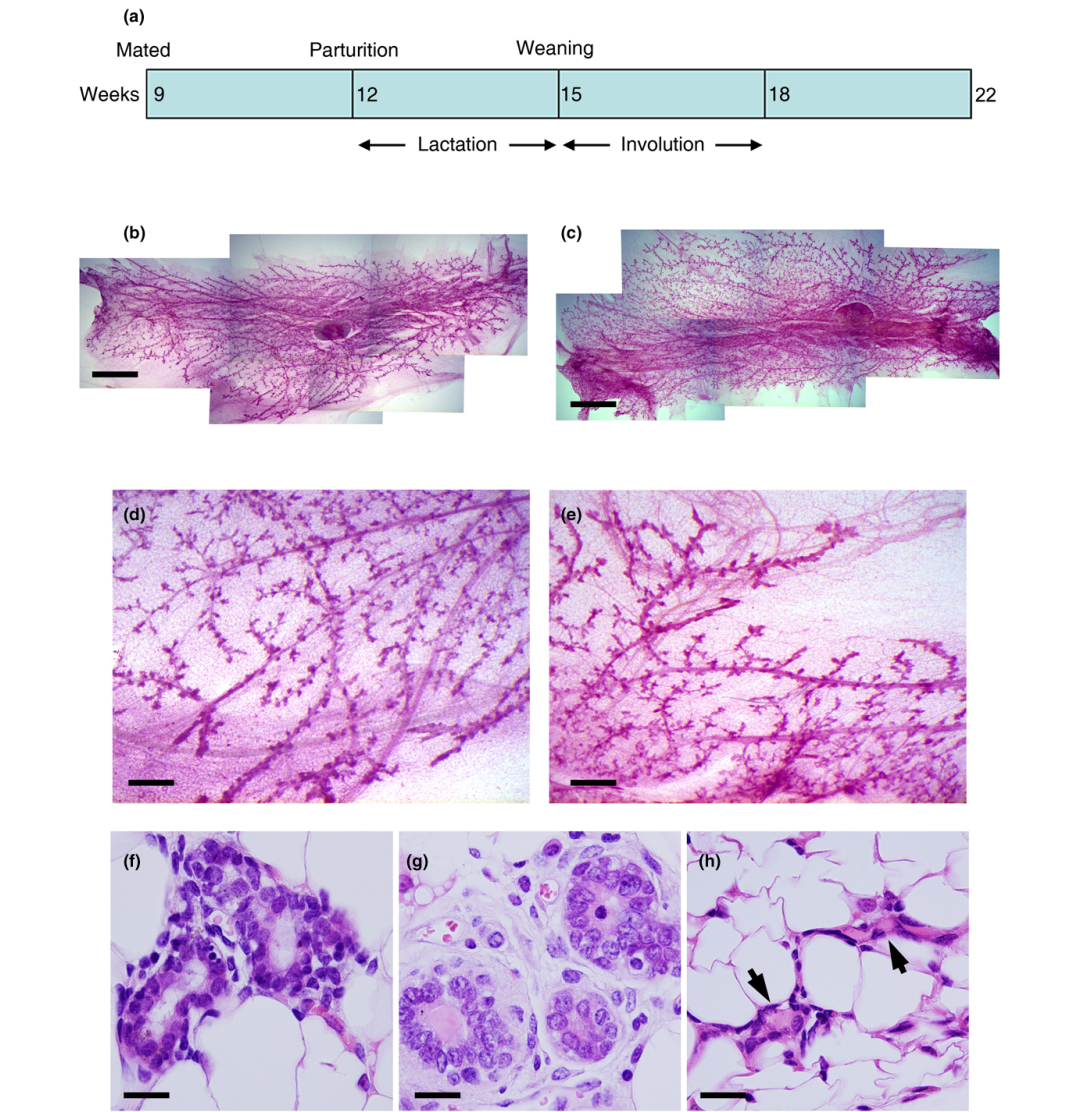
Seven-week post-lactation mouse mammary epithelium is fully regenerated and similar to age-matched virgin tissue. **(a) **Outline of experimental design. Mice were mated at 9 weeks of age, went through a normal pregnancy and nursed pups for 3 weeks. Involution occurred over an approximately 3-week period after pups were removed. After a further 4 weeks, when the animals were in total 22 weeks old, tissue was harvested. **(b), (d) **Carmine-stained whole mount of age-matched virgin (AMV) fourth mammary fat pad. **(c), (e) **Carmine-stained whole mount of parous fourth mammary fat pad. (b), (c) Bar = 3 mm. (d), (e) Bar = 750 μm. **(f) **H & E-stained section of AMV fourth mammary fat pad. Bar = 20 μm. **(g) **H & E-stained section of parous (7-week post-lactation) fourth mammary fat pad. Bar = 20 μm. **(h) **H & E-stained section of parous (3-week post-lactation) fourth mammary fat pad showing collapsed ducts (arrowheads). Bar = 20 μm.

All animal work was carried out under UK Home Office project and personal licences following local ethical approval and in accordance with local and national guidelines. Parous mice were obtained by mating 9-week-old FVB females and allowing normal parturition, lactation and weaning of pups (at 21 days post partum). The parous animals were then left to undergo mammary gland involution and remodelling for a further 7 weeks. Tissue was harvested for transplant at 22 weeks of age (Figure 1a). AMV FVB females were used as controls.

### Isolation, staining and flow cytometric analysis of primary mouse mammary cells

The procedure for isolation of primary mouse mammary cells, their staining and separation by flow cytometry has been previously described in detail [26]. In brief, fourth (abdominal) mouse mammary fat pads from FVB mice were processed by mechanical and enzymatic dissociation to liberate single cells. Cells were stained with anti-CD24-FITC (clone M1/69, 0.5 μg/ml; BD Biosciences, Oxford, UK), anti-CD45-PE-Cy7 (clone 30-F11, 0.25 μg/ml; BD Biosciences), anti-Sca-1-PE (clone D7, 0.5 μg/ml; BD Biosciences) and anti-CD49f-PE-Cy5 (clone GoH3, 5 μl/10^6 ^cells; BD Biosciences). To standardise gating on CD49f^High ^mammary epithelial cells, we used 5% interval linear density contour plots (see Additional data file 1) and considered the outer edge of the main body of the CD24^+/Low ^cells, as defined by the contour plots, to be the limit above which cells can be considered CD49f^High^.

For analysis of green fluorescent protein (GFP)-expressing cells isolated from transplanted fat pads, anti-CD24-FITC was replaced with anti-CD24-PE-Cy5 (clone M1/69, 0.6 μg/ml; eBioscience, Insight Biotechnology Limited, London, UK) and the anti-CD49f-PE-Cy5 was not used.

Cells were sorted on a FACSAria (BD Biosciences) equipped with violet (404 nm), blue (488 nm), green (532 nm), yellow (561 nm) and red (635 nm) lasers. Both cell sample and collection tubes were maintained at 4°C. Dead cells, CD45^+ ^leukocytes and nonsingle cells were excluded as described [26]. There was no difference in viability of parous and AMV cells detectable during sorting.

### Lentivirus production

Viral supernatants were generated by co-transfection of the expression vector pWPI and two packaging vectors (psPAX2 and pMD2.G) (Tronolab [35]) into HEK293T cells. Cells were re-fed with fresh medium (DMEM; Invitrogen, Paisley, UK) plus 10% FCS (PAA Laboratories, Yeovil, UK) after 24 hours. Supernatants were harvested 48 and 72 hours after transfection and were checked for absence of replication-competent virus. Supernatants were stored at -80°C until use.

### Mammary epithelial cell transduction and transplantation

Freshly isolated primary mouse mammary epithelial cells were resuspended at 1 × 10^6 ^cells/ml in viral supernatant and plated at 1 ml/well in ultra-low attachment 24-well plates (Corning, Fisher Scientific, Loughborough, UK) [36]. After 16 hours, the cells (now in clumps) were washed and replated in 1:1 DMEM:Ham's F12 medium (Invitrogen) with 10% FCS, 10 μg/ml insulin (Sigma, Poole, UK), 100 ng/ml epidermal growth factor (Sigma) and 10 ng/ml cholera toxin (Sigma) (growth medium) [37] and were transferred to ultra-low attachment six-well plates (Corning), usually combining the contents of four wells of the 24-well plates into one well of a six-well plate. After a further 24 hours, all of the cells were pooled, washed in PBS and resuspended in a volume of serum-free Leibowitz L15 medium (Invitrogen) such that entire volume of cells would be evenly distributed among all the cleared fat pads to be injected (for instance, given that the maximum injection volume per fat pad was 10 μl, if 10 fat pads were to be injected then the cells were resuspended in 100 μl). This ensured that the maximum number of cells per fat pad would be transplanted in order to guarantee successful outgrowths for subsequent analysis. The cells were injected into cleared fat pads as described [26] and were analysed after 8 weeks by mechanical/enzymatic digestion and flow cytometry as described above.

To estimate viral transduction efficiency, an aliquot of transduced cells was taken from those being used for the transplants and was maintained in culture for 1 week to allow time for expression of the GFP (which is not expressed immediately after transduction, making it difficult to estimate the transduction efficiency directly prior to transplantation). The percentage of GFP-positive cells was then measured either by flow cytometry or direct visualisation of cells cultured on coverslips. Using these methods, transduction efficiencies of between 40% and 90%, depending on the experiment, were determined.

### Limited dilution cleared fat pad transplantation of mammary cells to determine stem/progenitor cell frequencies

Analysis of stem/progenitor cell capacity of mammary cell populations by limited dilution transplantation into cleared mammary fat pads has been previously described in detail [11,26]. Primary mammary epithelial cells were freshly isolated from mouse mammary glands and immediately stained and flow sorted (with no intervening culture period). Using the flow cytometry gates defined by analysis of GFP-transduced mammary epithelial cells, purified total epithelial cells (CD24^+/Low ^Sca-1^- ^cells plus the total CD24^High ^population) were isolated. The cells were immediately counted and resuspended in serum-free L15 medium at varying concentrations (for instance, 20,000, 10,000 or 5,000 cells in 10 μl, the injection volume). The cells were then transplanted into cleared fat pads. They did not undergo any *in vitro *culture, unlike the cells used for viral transduction. After 8 weeks, the transplanted fat pads were whole-mounted and carmine-stained to determine the number and extent of any successful outgrowths. The proportion of stem/progenitor cells was determined using the L-Calc limiting dilution statistical analysis program [27].

### Keratin 14/keratin 18/ER immunofluorescence staining of paraffin sections

Antigen sites were retrieved by microwaving in 0.01 M citrate buffer, pH 6. Nonspecific immunoreactivity was blocked with MOM mouse immunoglobulin blocking reagent (stock MOM immunoglobulin blocking reagent in 250 μl Tris-buffered saline; Vector Laboratories, Peterborough, UK) and 30 minutes in DAKO protein block (DAKO, Ely, UK). Sections were incubated with antibodies against keratin 14 (mouse IgG_3 _clone LLOO2, 1:500 dilution; Abcam, Cambridge, UK) and keratin 18 (mouse IgG_1 _clone Ks18.04, 1:2 dilution; Progen, Heidelberg, Germany) or keratin 14 and ER (mouse IgG_1 _clone 1D5, 1:40 dilution; DAKO) overnight at 4°C. After washing in 0.05% Tween in Tris-buffered saline, sections were stained with goat anti-mouse IgG_3_-Alexa488 (1:500 dilution; Invitrogen) and IgG_1_-Alexa555 (conjugated in-house according to the manufacturer's instructions using Sigma anti-mouse IgG_3 _antibody and the AlexaFluor-555 protein labelling kit; Invitrogen). Sections were washed, counterstained with DAPI and mounted in Vectashield (Vector Laboratories).

All sections were examined on a TCS SP2 confocal microscope with an Acousto-Optical Beam Splitter and lasers exciting at 405 nm, 488 nm and 555 nm (Leica Microsystems, Milton Keynes, UK). Multicolour images were collected sequentially in three channels and captured using the Leica system and Leica TCS image acquisition software. Co-localisation overlays were generated using TCS software. Single antibody-stained control sections either lacking the first antibody or in which the primary antibody was combined with an inappropriate second antibody were used to confirm lack of nonspecific staining and cross-reactivity between secondary and primary antibodies.

## Results and discussion

### Seven-week post-lactation mice have fully regenerated the mammary epithelium

The strategy for preparing 7-week post-lactation (hereafter termed parous) mice is shown diagrammatically in Figure 1a. To confirm that these animals had completely regenerated their mammary epithelium and were structurally similar to AMV animals, whole-mount analysis and histological examination of mammary fat pads from mice taken at the same point in the oestrus cycle (at oestrus) were compared.

On the basis of these analyses, the mammary epithelium from parous animals had completely regenerated following post-lactational involution and at a gross morphological level was very similar to that of AMV animals (Figure 1b to 1e). H & E sections of parous and AMV glands showed that both had well-defined ducts and alveolar structures although there were differences in nuclear morphology between them (Figure 1f, g). In contrast to the well-defined ducts of the AMV and parous animals, sections through fat pads isolated from animals at 3 weeks post lactation showed that the tissue contained many collapsed ducts that had not yet returned to the normal resting morphology (Figure 1h).

### Mammary cells from parous and AMV animals have similar flow cytometry profiles

We have previously used CD24 and Sca-1 reactivity to purify epithelial cells from virgin mouse mammary tissue [11]. Basal cells were CD24^+/Low ^Sca-1^- ^and consisted mainly of myoepithelial cells but also included the stem cells, which could be further enriched by sorting for α_6 _integrin (CD49f) expression, supporting a previous report [27]. The luminal cells were CD24^+/High ^and could be divided in to CD24^+/High ^Sca-1^- ^ER^- ^and CD24^+/High ^Sca-1^+ ^ER^+^. To determine whether parity might change the CD24 Sca-1 CD49f flow cytometry profile of mammary cells and, in particular, whether changes might occur to the CD24^+/Low ^Sca-1^- ^CD49f^High ^stem-cell-enriched population, mammary cell preparations from parous and AMV animals were stained for expression of these markers and were analysed by flow cytometry.

**Figure 2:**
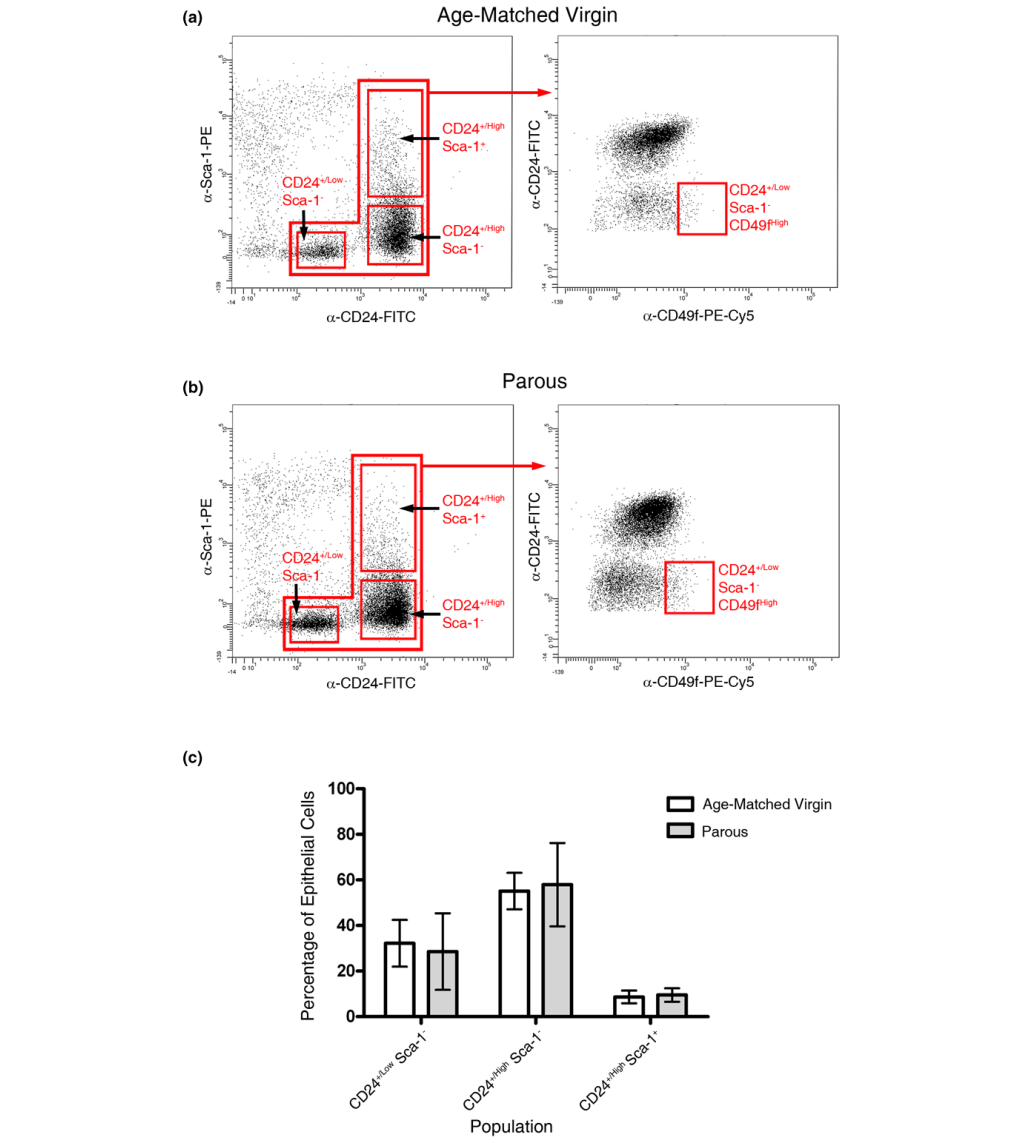
CD24 and Sca-1 flow cytometry isolates similar populations in age-matched virgin and parous tissues. **(a) **Age-matched virgin (AMV) cells and **(b) **parous cells were stained with antibodies against CD24, Sca-1 and CD49f. The CD24 and Sca-1 plots (left panels) identified CD24^+/Low ^Sca-1^-^, CD24^+/High ^Sca-1^- ^and CD24^+/High ^Sca-1^+ ^cells in both AMV and parous cell preparations. Analysis of these populations for CD49f expression (right panels) enabled identification of the CD24^+/Low ^Sca-1^- ^CD49f^High ^population previously shown to be enriched for mammary stem cells [11,26,27]. See also Additional data file 1. **(c) **Relative abundance of CD24^+/Low ^Sca-1^-^, CD24^+/High ^Sca-1^- ^and CD24^+/High ^Sca-1^+ ^cells as a percentage of the total epithelium in AMV and parous tissue.

The analysis results (Figure 2) showed that there were no significant differences between the cell preparations. In particular, the percentage of CD24^+/Low ^Sca-1^- ^CD49f^High ^cells as a percentage of the total epithelium in the parous animals was 1.50 ± 0.85% (mean ± standard deviation; n = 3 independent preparations), and in the AMV animals the percentage was 1.37 ± 0.49% (n = 3 independent preparations). There were also no significant differences between the proportions of CD24^+/Low ^Sca-1^- ^basal cells, CD24^+/High ^Sca-1^- ^luminal ER^- ^cells and CD24^+/High ^Sca-1^+ ^luminal ER^+ ^cells in the parous animals compared with AMV animals (Figure 2c). Consistent with these data, Siwko and colleagues noted no differences between the proportion of CD24^+ ^CD29^High ^cells (essentially equivalent to CD24^+/Low ^CD49f^High ^[38]) in virgin mice compared with parous mice [23].

As there were differences between the nuclei in sections of parous mice compared with AMV animals, the flow cytometry profiles were analysed for differences in forward or side scatter. Mean scatter values of epithelial cells were normalised to the mean scatter of the lymphocytes within each sort. The data (from three independent parous and three independent AMV sorts; see Additional data files 2 and 3) showed that both the CD24^+/High ^luminal populations had similar forward scatter values, whereas the CD24^+/Low ^Sca-1^- ^basal cells had significantly smaller forward scatter values than both luminal populations in the AMV tissue (*P *< 0.05, *t*-test on log_10_-transformed data), suggesting they are smaller than the luminal cells. Changes in forward scatter with parity were negligible, with only the forward scatter of the basal cell population showing a significant (*P *< 0.05, *t*-test on log_10_-transformed data) increase in parous tissue, suggesting a slight increase in mean size. There were no significant differences in side scatter between the different populations in either the virgin or the parous animals and no significant side scatter differences in parous cells compared with AMV cells.

It has been reported that the p53 function and apoptotic activity are increased in the parous mammary epithelium [39]. To determine whether there were viability differences between AMV and parous tissue, therefore, cell samples that had been sorted (excluding DAPI^+ ^dead cells) and then immediately resorted to check for purity were analysed for post-sort viability. There were no significant viability differences between the tissues, with the AMV cells having a post-sort viability of 78.56 ± 1.70% (n = 5 independent sorts) and the parous cells a post-sort viability of 81.63 ± 3.63% (n = 4 independent sorts).

### Total mammary epithelium can be reliably purified from mouse mammary tissue

To directly address the issue of whether parity changed the proportion of cells in the mammary epithelium with stem/progenitor cell capacity, a series of limiting dilution transplant experiments was carried out comparing the ability of cells isolated from parous animals and AMV animals to repopulate a cleared mouse mammary fat pad. First, we determined that we could purify the entire mammary epithelial cell population from mammary fat pads using CD24 and Sca-1 staining. Such a sorting strategy would ensure that the number of stem/progenitor cells as proportion of the total epithelium only would be tested by limiting dilution transplants, and the results would not be biased by parity-dependent changes in numbers of nonepithelial cells within the gland. Furthermore, as unequivocally pure basal stem cells cannot yet be isolated, and as progenitors with some transplant activity can be identified in other populations [11], any approach comparing the transplantation ability of a single epithelial subpopulation (for example, CD24^+/Low ^Sca-1^- ^CD49f^High ^cells) from two different developmental stages may be biased by changes in the purity of stem cells within the population or changes in the numbers of progenitors in other populations. Testing the total purified epithelium ensures that all cells with potential stem/progenitor activity will be assayed.

**Figure 3:**
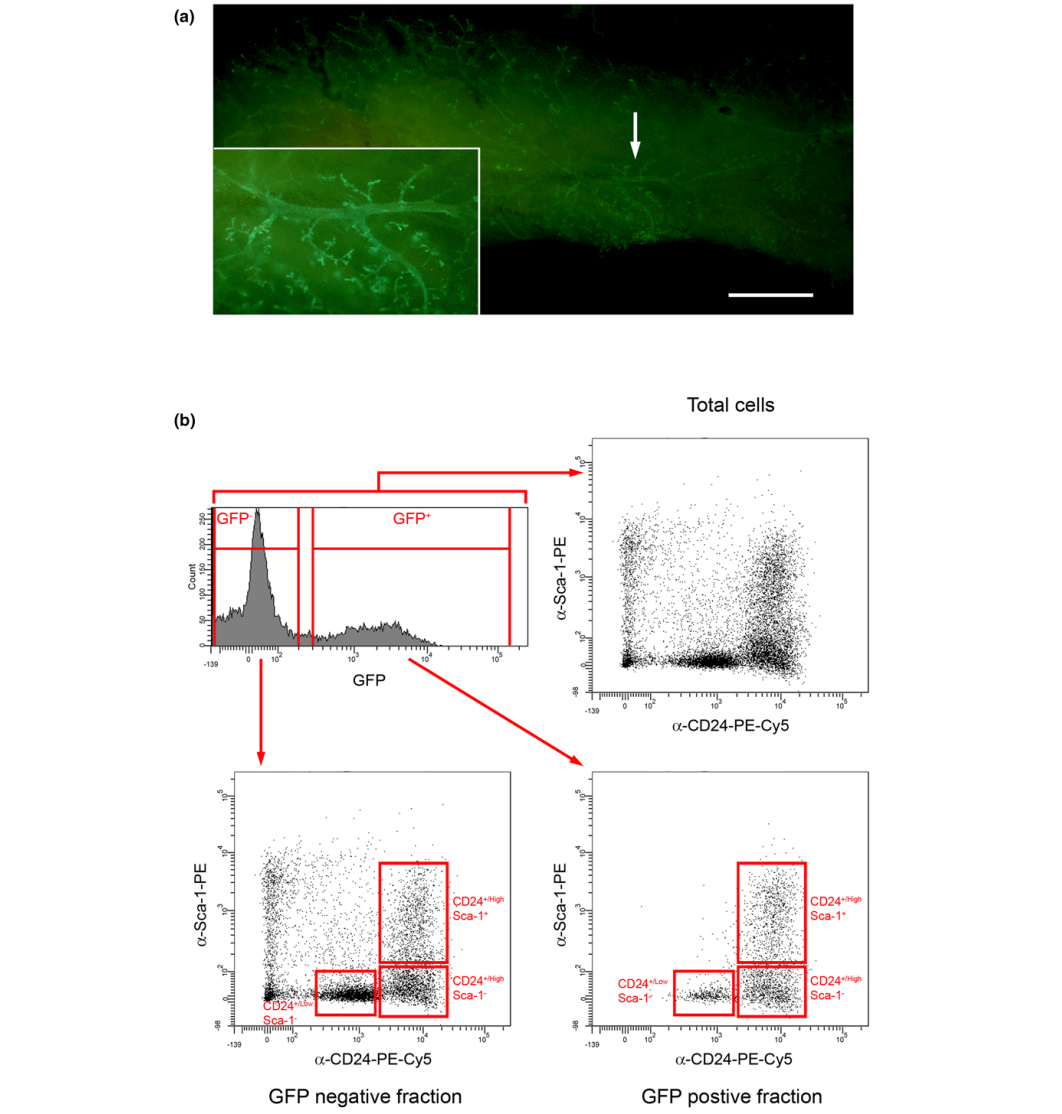
Isolation of total epithelial cell populations from mammary epithelium. **(a) **Whole mount of mammary fat pad transplanted with primary cells transduced with green fluorescent protein (GFP) lentivirus. Arrow, area enlarged in inset. Bar = 2.5 mm. **(b) **Flow cytometric analysis of transplanted epithelium. The histogram indicates levels of GFP fluorescence in cells isolated from transplanted mammary fat pads. The CD24 Sca-1 staining pattern of the total cells (GFP^- ^and GFP^+^) harvested from the fat pads is shown. The CD24 Sca-1 staining patterns of the GFP^- ^and GFP^+ ^cells are also shown separately. GFP^+ ^cells, which must represent the progeny of transduced stem cells, are found only in the CD24^+/Low ^Sca-1^-^, CD24^+/High ^Sca-1^- ^and CD24^+/High ^Sca-1^+ ^regions. Data are representative of four independent experiments.

To confirm that we had identified the CD24 and Sca-1 staining profile that defined the entire epithelium, primary mammary cells were transduced with a lentivirus expressing GFP and then transplanted into cleared fat pads. Fat pads were examined after 8 weeks and were found to contain extensive GFP^+ ^outgrowths (Figure 3a). Successful repopulation of cleared fat pads is a property of mammary stem/progenitor cells and results in the generation of a mammary epithelial outgrowth containing all of the mammary epithelial cell types [11]. The GFP^+ ^outgrowths must therefore have been derived from virus-transduced mammary stem/progenitor cells and would be expected to contain the full range of mammary epithelial cells labelled with GFP. To test this hypothesis, the GFP^+ ^outgrowths were digested to single cells, stained for CD24 and Sca-1 expression and analysed by flow cytometry (Figure 3b). GFP^- ^cells, which would include cells derived from the host fat pads and transplanted epithelium derived from stem/progenitor cells that had not been transduced with virus, were found to be either CD24^-^, CD24^+/Low ^or CD24^+/High ^and either Sca-1^- ^or Sca-1^+^. GFP^+ ^cells, however, were found only in the combined CD24^+/Low ^Sca-1^-^, CD24^+/High ^Sca-1^- ^and CD24^+/High ^Sca-1^+ ^regions. This confirmed that a flow cytometry sort gate over these combined areas defined the total mammary epithelium.

### Cleared fat pad transplant analysis demonstrates that stem/progenitor activity of parous and AMV tissue does not differ significantly

**Figure 4:**
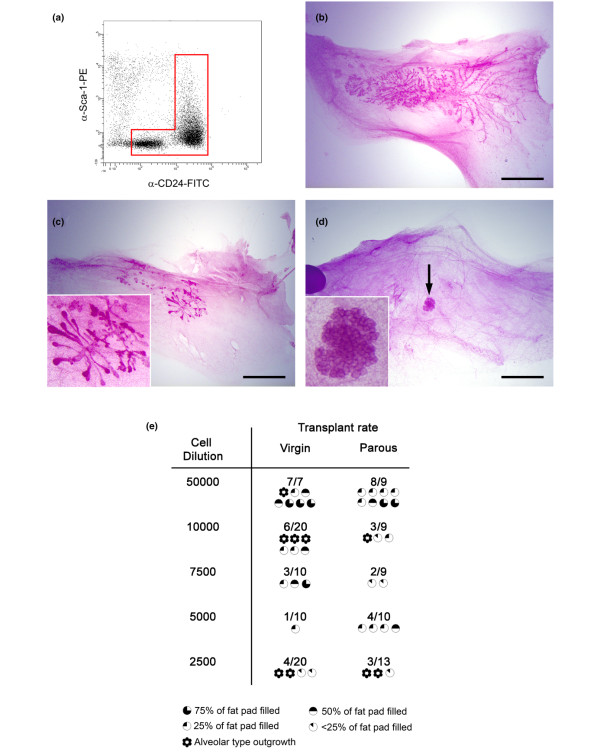
Proportion of stem/progenitor cells in mammary epithelium unchanged in parous compared with age-matched virgin tissue. **(a) **Flow cytometry plot of cells isolated from age-matched virgin (AMV) tissue and stained with anti-CD24 and anti-Sca-1 antibodies showing a typical gate used to isolate total mammary epithelial cells for transplantation. This gate was based on the data obtained from the lentiviral transduction experiments, which demonstrated that the CD24^+/Low ^Sca-1^- ^plus the total CD24^+/High ^regions defined the entire mammary epithelium. **(b) to (d) **Carmine stained whole mounts showing examples of (b) large ductal outgrowths, (c) small ductal outgrowths and (d) an alveolar structure generated by transplantation. Arrow, region enlarged in the inset. Bar = 4 mm. **(e) **Table of results of limiting dilution transplants indicating the number of outgrowths obtained and the number of fat pads transplanted for each cell dilution. The extent to which each outgrowth filled the host fat pad is indicated by the symbols below the numbers. Data are from seven independent cell isolation and transplant sessions.

Having established that the total mammary epithelium could be isolated, limiting dilution transplants with total epithelial cells (Figure 4a) derived from parous and AMV animals were carried out. The cells were freshly isolated and did not undergo any *in vitro *culture period prior to transplantation. Transplants were examined after 8 weeks (examples of outgrowths are shown in Figure 4b to 4d) and the number of outgrowths and the extent to which they filled the mammary fat pads were estimated (Figure 4e). The majority of successful transplants consisted of a spreading ductal network (Figure 4b, c) with secondary branching and alveolar bud-like structures in the larger outgrowths. A number of outgrowths, however, appeared to consist of alveolar-type structures only with no ductal component (Figure 4d).

**Figure 5:**
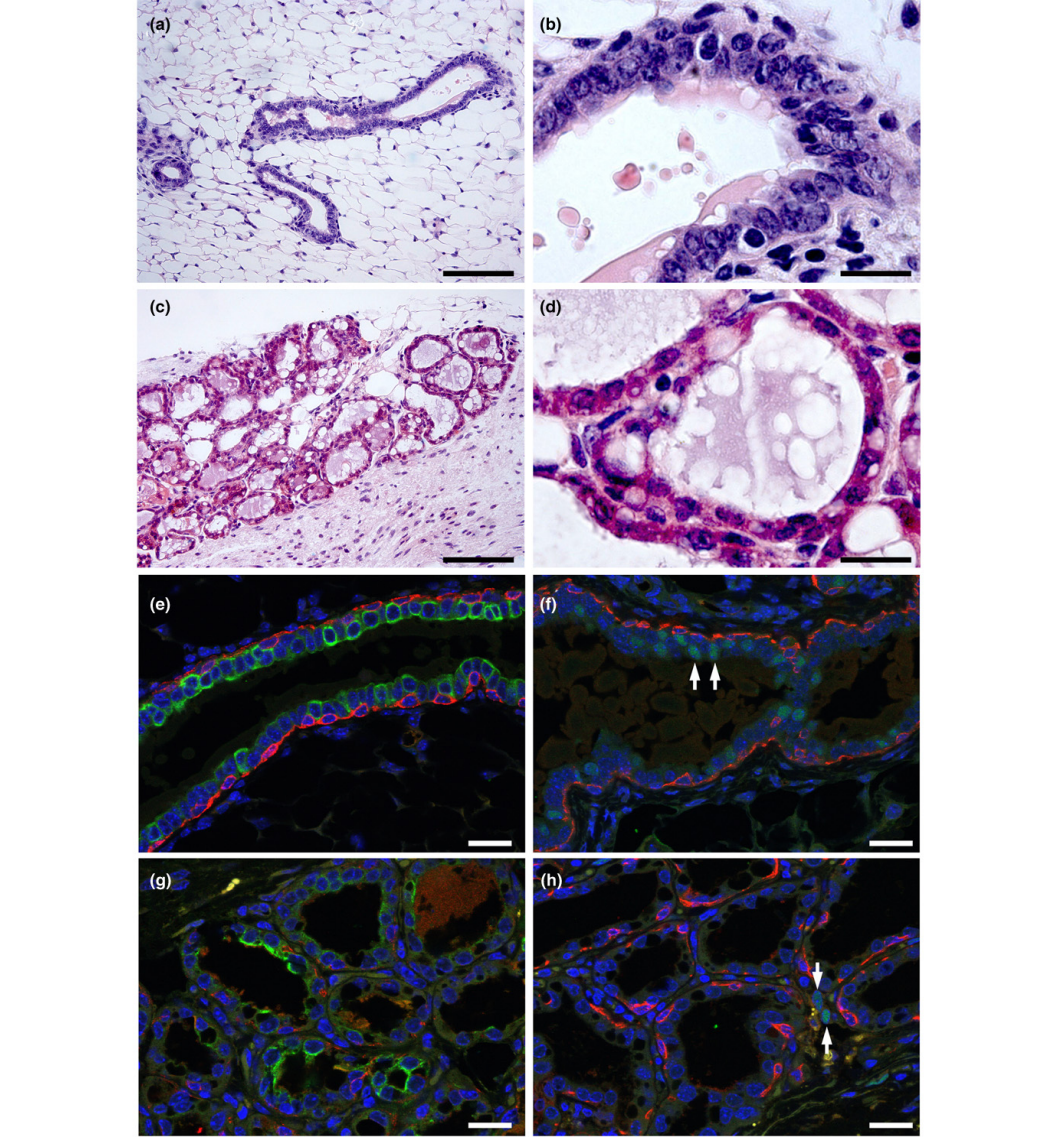
Outgrowths of transplanted parous and age-matched virgin tissue.  Outgrowths of transplanted parous and age-matched virgin tissue contain basal/myoepithelial cells, luminal oestrogen receptor-negative (ER^-^) cells and luminal oestrogen receptor-positive (ER^+^) cells. **(a), (b) **H & E-stained section of ductal-type transplant outgrowth. **(c), (d) **H & E-stained section of alveolar-type transplant outgrowth. (a), (c) Bar = 120 μm. (b), (d) Bar = 20 μm. **(e) to (h) **Sections of outgrowth stained by multiple immunofluorescence for cell-type specific markers. All nuclei are counterstained with DAPI (blue). Bar = 20 μm. (e) Ductal outgrowth stained for keratin 14 (red) and keratin 18 (green). (f) Ductal outgrowth stained for keratin 14 (red) and ER (green). Arrows, examples of ER^+ ^luminal epithelial nuclei. (g) Alveolar outgrowth stained for keratin 14 (red) and keratin 18 (green). (h) Alveolar outgrowth stained for keratin 14 (red) and ER (green). Arrows indicate rare interstitial ER^+ ^cells between alveolar structures.

Small pieces of tissue were dissected out from all outgrowths and sectioned to confirm their identity (Figure 5a to 5d). Immunostaining of sections through outgrowths for cell-type-specific markers (keratin 14, keratin 18 and ER) confirmed that all ductal-type outgrowths (derived from both parous and AMV animals) included myoepithelial, luminal ER^- ^and luminal ER^+ ^cells (Figure 5e, f). In the alveolar-type transplants, however, although keratin 18-positive cells could easily be detected (Figure 5g), the number of keratin 14-positive cells was variable (compare Figure 5g with Figure 5h) and ER^+ ^cells could not be observed in the epithelium. They could be observed, however, in interstitial cells between the alveoli (Figure 5h, arrows). Again, these findings were similar in both parous and AMV animals.

The rate of successful transplants was used to estimate the proportion of stem/progenitor cells in each population using the L-Calc analysis package [27]. The calculations showed that the AMV animals had a mammary epithelial stem/progenitor frequency of 1 in 20,636 cells (95% confidence limits: 1 in 13,157 to 1 in 32,366) and the parous animals had a mammary stem/progenitor frequency of 1 in 18,538 cells (95% confidence limits: 1 in 11,238 to 1 in 30,580). Stem/progenitor cell frequencies in the AMV cells and the parous cells were therefore not significantly different following a single pregnancy in the mature virgin animal. However, it is possible that multiple pregnancies and/or increasing the time at which cells are harvested for assay beyond 7 weeks after weaning may still alter stem cell numbers following the initial first pregnancy at 9 weeks of age. Future studies will address these issues.

The alveolar-only outgrowths did not contain cells with detectable ER and may have arisen from progenitors with a limited differentiation potential that did not include the ER^+ ^cell lineage. Such outgrowths would therefore be incapable of responding to oestrogen and initiating ductal elongation. A hierarchy of stem/progenitor cells including ductal–alveolar progenitors capable of generating the entire mammary epithelium, ductal progenitors that can generate only ducts and alveolar progenitors that can generate only alveoli has been previously suggested [30]. Our results support such a hierarchy and suggest that its underlying basis is the cell lineage differentiation potential of the outgrowth initiating cells. Whether such alveolar-only progenitors should be included in calculations to determine stem/progenitor frequencies, however, is unclear. We therefore recalculated the stem/progenitor frequencies excluding the transplants that resulted in alveolar outgrowths from the calculations. Excluding these transplants, the stem/progenitor cell frequency in the AMV animals was 1 in 33,786 cells (95% confidence limits: 1 in 20,152 to 1 in 56,644) and in the parous animals was 1 in 23,097 cells (95% confidence limits: 1 in 13,762 to 1 in 38,766), suggesting a trend towards an increasing number of stem/progenitor cells specifically with ductal outgrowth potential in the parous tissue. The difference, however, is not statistically significant [40].

To our knowledge, the presence of alveolar-type outgrowths with no ductal component in mammary fat pad transplants has only been reported twice previously, once by us [41] and once by Smith in the original report describing the phenomenon [30]. Smith reported that out of 22 transplants from limiting dilutions (5,000 per fat pad) of mammary epithelial cells, 10 were of the alveolar-only type (Smith terms these lobular). In the current report, eight out of 22 transplants from the virgin donors formed alveolar outgrowths, comparable with Smith's data [30]. In our previous report, four out of five outgrowths derived from mammary side population cells had the alveolar phenotype [41]. In both of these previous reports, transplant host animals were mated prior to whole mounting of fat pads – and this may have influenced formation of alveolar structures. In the current report, however, animals were not mated prior to whole mounting, yet many alveolar type outgrowths were still observed. In our previous study, the donor animals were 8-week-old to 10-week-old virgin FVB mice and the use of side population sorting may have enriched for alveolar progenitors [41,42]. Smith does not make the age of the donor mice clear [30]; in the current study, the donor mice were 22 weeks old. In our previous transplantation studies in which cells from 10-week-old virgin FVB mice were transplanted but the animals were not mated prior to whole mounting, no alveolar-only type outgrowths were observed [11,26]. It is therefore possible that two factors influence the development of alveolar-only type structures in cleared fat pad transplantation – namely increasing age of the donor mice, and induction of pregnancy in the host mice prior to whole-mount analysis. Further transplant experiments will be needed to clarify this issue.

We chose to use flow cytometry based on CD24 and Sca-1 expression profiles to purify the total epithelial cell compartment of parous and AMV mammary epithelium for transplantation. The benefits of this approach are that it allows transplantation of a well-defined cell population, it enables comparison with our previous data [11,26] and it permits stem/progenitor cell numbers to be measured as a fraction of the epithelium without possible confounding effects of co-transplantation of stromal cells that may enhance or suppress transplantation as a result of their own responses to parity. We (and others) have previously shown that stem/progenitor cells in the virgin mouse mammary epithelium are CD24^+ ^[11,24,26,27] and we used GFP-marking and transplantation to confirm that we could isolate the total mammary epithelium with our flow sorting strategy. We have only directly demonstrated this, however, in the virgin animal. We cannot definitively exclude the possibility that this sorting strategy may exclude some stem/progenitor cells in the parous tissue. Furthermore, the use of purified epithelial populations, rather than mixed cell isolates, may alter estimates of stem/progenitor cell potential.

Siwko and colleagues estimate stem/progenitor cell frequencies in the unsorted epithelial cell fractions of AMV and parous tissue as 1 in 2,608 and 1 in 5,050 cells, respectively. This is in agreement with a previous estimate of stem/progenitor cell frequency in unsorted mammary tissue of approximately 1 in 1,400 cells [27] and our own estimates of the frequency of stem/progenitor cells in unsorted mammary cell isolates of 1 in 2,951 cells (H Kendrick and M Smalley, unpublished data). These contrast, however, with our estimated stem cell frequency in the purified AMV epithelial cells of 1 in 20,636 cells. We previously published a limiting dilution transplant series of basal epithelial cells, luminal ER^- ^cells and luminal ER^+ ^cells isolated by flow sorting. These series gave estimated stem cell frequencies in the three populations of 1 in 3,480 for the basal cells, 1 in 14,317 for the luminal ER^- ^cells and 1 in 56,614 for the luminal ER^+ ^cells [11]. These estimates, from epithelial populations enriched (basal) or depleted (luminal) for stem cell activity, are fully consistent with the current estimate of 1 in 20,636 cells from the total virgin epithelium and suggest that cell purification lowers the ability of transplanted cell isolates to generate outgrowths. It may be that transplanting of total mixed mammary cells enhances the rate of successful transplants because of co-transplant of nonepithelial cells that act to stimulate the outgrowth of the stem cells. Alternatively, it may be that the sorting procedure itself is deleterious. Potentially, both factors may be relevant. Importantly, however, this does not mean that the differences between outgrowth potential are due to differential viability between the cells [11].

It should also be noted that transplantation at limiting dilution into cleared fat pads is forcing stem/progenitor cells into a behaviour they would not normally perform physiologically during routine tissue turnover or pregnancy-dependent epithelial expansion. This may result in failure of some genuine stem cells to form outgrowths or may allow more differentiated progenitors to reacquire primitive stem cell-like features and generate outgrowths, as has been recently reported for differentiating spermatogonial progenitors [43]. Nevertheless, it is still the case that the relative cleared fat pad transplant potential of the different mammary epithelial populations reflects the relative enrichment or depletion of stem cells. Transplantation experiments first suggested that mammary epithelial stem cells were most strongly enriched in the basal cell layer [11,24,26,27], and this was confirmed by the recent report from Taddei and colleagues, who knocked out the stem cell marker β_1_-integrin in the basal cell layer of the mammary epithelium in mice [28]. This blocked mammary stem cell function and gave a phenotype that included ductal outgrowth defects in the virgin mice. Knockout of β_1_-integrin in alveolar progenitors [32] did not affect the virgin tissue but did affect pregnancy-dependent development [32,44]. Therefore, although cell separation and transplantation experiments do have potential limits, the results from them are supported by data from the intact animal.

Siwko and colleagues have recently demonstrated that pregnancy results in a twofold reduction in stem cell numbers, a conclusion apparently at odds with the findings we present here. There are, however, three main differences between the studies. First, Siwko and colleagues used fewer animals as donors and did not keep them at a consistent age when tissue was harvested. We used larger pools of animals for each harvest (at least 10 mice for each experiment) and all were at the same timepoint, as illustrated in Figure 1a. Second, the transplants of Siwko and colleagues were carried out with unsorted total mammary cells. This probably explains the overall difference in take rate (1 in 2,608 for virgin cells from Siwko and colleagues, and 1 in 20,636 for virgin cells from our data; see discussion above). Third, and most important, Siwko and colleagues mated their mice at 5 weeks of age, early in puberty, whereas we mated our mice at 9 weeks of age when the mammary epithelium is essentially fully matured.

Although these results will need to be confirmed by a future analysis in parallel, they suggest that pregnancy during puberty reduces stem/progenitor cell numbers, but a pregnancy occurring once the gland is fully developed does not. Considering the clear epidemiological data that an early pregnancy is protective against breast cancer, but the protective effect is gradually lost with increasing age prior to first full-term pregnancy [2], this suggests a relationship between changes in stem cell numbers and alterations in breast cancer risk. However, a direct functional link remains to be proven. If there is such a link, it is unlikely to be straightforward as mammary stem cells are ER^- ^[10,11] but pregnancy specifically protects against ER^+^PR^+ ^breast cancer. It is thought that normal tissue stem cells are important targets for tumourigenic change because of their long *in vivo *lifespan, and it has been suggested that different breast cancer subtypes have their origin in different stem/progenitor cell types [45-47]. It is possible that if the target for generation of ER^+^PR^+ ^tumours is an ER^+ ^progenitor, then a reduction in ER^- ^stem cell numbers may reduce the rate at which ER^+ ^progenitors are formed, reducing the size of the target population for transformation. Alternatively, as discussed above, oestrogen may have ER-independent effects on stem cells or the effects may be mediated through paracrine interactions with ER^+ ^differentiated cells.

In any case, it is highly likely that the alterations in the breast cancer risk profile caused by pregnancy are multifactorial in origin and include other mechanisms besides the potential for alteration to stem/progenitor cell populations [2]. Such mechanisms may include changes in oestrogen responsiveness [48-50] or gene expression [51-53] of cells, or cell subsets, within the mammary gland. Changes in the hormonal milieu of the body [48,54,55] have also been proposed to mediate the protective effects of parity, possibly through alterations in the stem cell niche [56]. Manipulation of hormone levels can mimic the protective effects of pregnancy [54,55], and administration of insulin-like growth factor 1 can re-establish the sensitivity of the parous mammary gland to carcinogenesis [57]. Future studies comparing changes in stem cell numbers in animals mated during puberty or at maturity should therefore include hormonal manipulations followed by treatment with carcinogens. This will enable assessment of whether changes in stem cell numbers in early versus late pregnancy do indeed correlate with parity-dependent resistance to carcinogenesis and, if they do, whether insulin-like growth factor 1 treatment not only removes the resistance but simultaneously increases stem cell numbers. Such data would strongly support a mechanistic connection between parity-mediated protection from ER^+^PR^+ ^breast cancer and changes in stem cell numbers as a result of early, but not late, pregnancy.

## Conclusions

There are no significant differences in stem/progenitor cell numbers between the mammary epithelium derived from mature adult mice (9 weeks old) that have been through a full-term pregnancy and lactation, followed by 7 weeks of involution and remodelling, compared with the mammary epithelium derived from AMV mice. In contrast, recent data have shown that animals mated at 5 weeks of age have a twofold reduction in stem/progenitor cell numbers [23]. Given the association between early, but not late, pregnancy and breast cancer risk reduction in humans, this finding is striking and suggestive – although a mechanistic connection between stem cell numbers and breast cancer risk remains to be established.

## Abbreviations

AMV: age-matched virgin; DAPI: 4,6-diamidino-2-phenylindole dihydrochloride; DMEM: Dulbecco's modified Eagle's medium; ER: oestrogen receptor; FCS: foetal calf serum; FITC: fluorescein isothiocyanate; GFP: green fluorescent protein; H & E: haemotoxylin and eosin; PBS: phosphate-buffered saline; PE: phycoerythrin; PR: progesterone receptor.

## Competing interests

The authors declare that they have no competing interests.

## Authors' contributions

KB contributed to the study design, isolated mammary cell populations for transplant, analysed transplanted tissues, developed immunostaining protocols, stained and analysed tissue sections and assisted with writing manuscript. HK carried out transplantations and analysed results of lentivirus transplants. JLR and GM assisted with transplantations and produced lentivirus. F-AM developed immunostaining protocols, and stained and analysed tissue sections. AA contributed to the study design and assisted with writing the manuscript. MJS contributed to the study design, analysed the results of transplants and wrote the manuscript.

## Supplementary Material

Additional file 1 An Adobe Photoshop TIF file containing an image that shows gating mammary epithelial cells for CD49 expression, the data from the CD24 CD49f flow cytometry plots in Figure 2 plotted as 5% linear density contour plots to delineate the main body of CD24^+/Low ^cells. The CD24^+/Low ^CD49f^High ^gate is set at the edge of the main body of cells. Click here for file

Additional file 2 An Adobe Photoshop TIF file containing an image that shows a comparison of normalised mean forward scatter/side scatter values for CD24^+/Low ^Sca-1^-^, CD24^+/High ^Sca-1^- ^and CD24^+/High ^Sca-1^+ ^mammary epithelial cells. The figure shows mean forward scatter (left-hand column) and side scatter (right-hand column) values normalised to lymphocyte forward scatter and side scatter values for the CD24^+/Low ^Sca-1^- ^(basal), CD24^+/High ^Sca-1^- ^(luminal ER^-^) and CD24^+/High ^Sca-1^+ ^(luminal ER^+^) populations isolated from AMV (top row) and parous (bottom row) mammary tissue (n = 3 independent sorts). Click here for file

Additional file 3 An Adobe Photoshop TIF file containing an image that shows changes in normalised mean forward scatter/side scatter values of mammary epithelial cell subpopulations between parous and AMV mammary tissue. The figure shows mean forward scatter and side scatter values normalised to lymphocyte forward scatter and side scatter values in AMV and parous tissue for the CD24^+/Low ^Sca-1^- ^(basal; top), CD24^+/High ^Sca-1^- ^(luminal ER^-^; middle) and CD24^+/High ^Sca-1^+ ^(luminal ER^+^; bottom) populations (n = 3 independent sorts). Click here for file
