# Supplementary figures and images for: Pregnancy in the mature adult mouse does not alter the proportion of mammary epithelial stem/progenitor cells
Source: Breast Cancer Res. 2009 Apr 23;11(2):R20. doi: 10.1186/bcr2245 (PMC2688949; doi:10.1186/bcr2245)

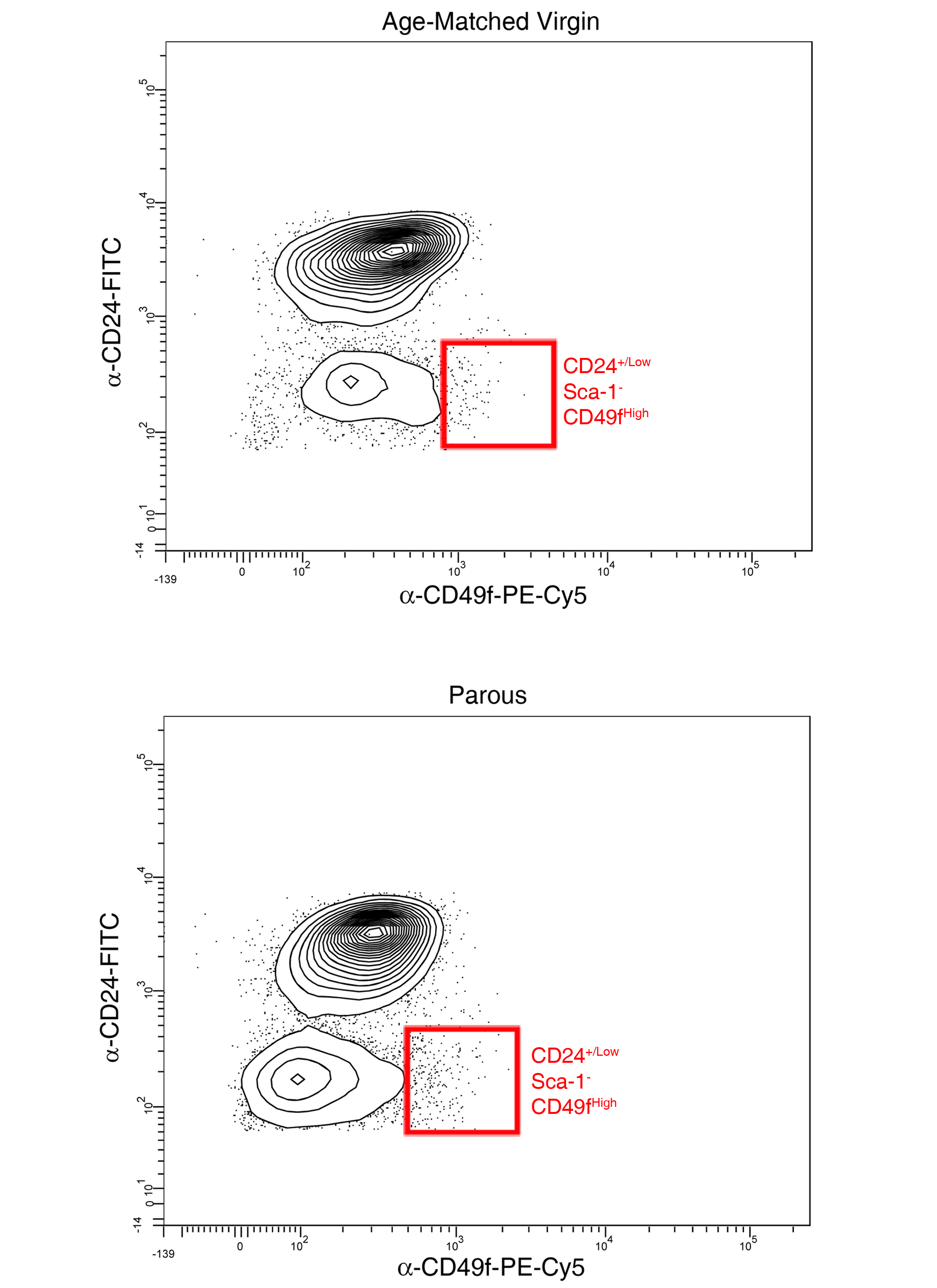

Supplement: Additional file 1 — An Adobe Photoshop TIF file containing an image that shows gating mammary epithelial cells for CD49 expression, the data from the CD24 CD49f flow cytometry plots in Figure 2 plotted as 5% linear density contour plots to delineate the main body of CD24+/Low cells. The CD24+/Low CD49fHigh gate is set at the edge of the main body of cells. [file bcr2245-S1.tiff]

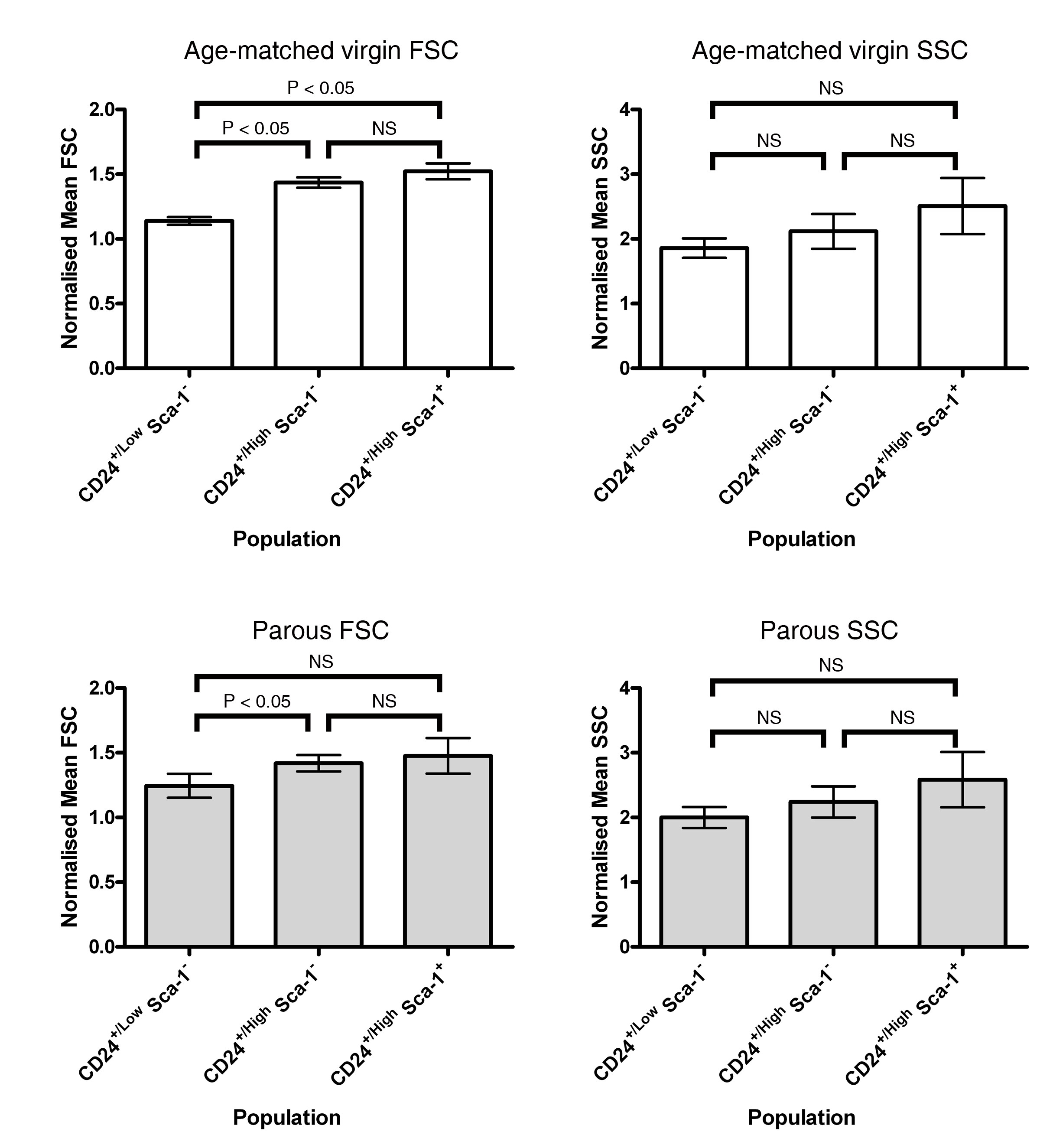

Supplement: Additional file 2 — An Adobe Photoshop TIF file containing an image that shows a comparison of normalised mean forward scatter/side scatter values for CD24+/Low Sca-1-, CD24+/High Sca-1- and CD24+/High Sca-1+ mammary epithelial cells. The figure shows mean forward scatter (left-hand column) and side scatter (right-hand column) values normalised to lymphocyte forward scatter and side scatter values for the CD24+/Low Sca-1- (basal), CD24+/High Sca-1- (luminal ER-) and CD24+/High Sca-1+ (luminal ER+) populations isolated from AMV (top row) and parous (bottom row) mammary tissue (n = 3 independent sorts). [file bcr2245-S2.tiff]

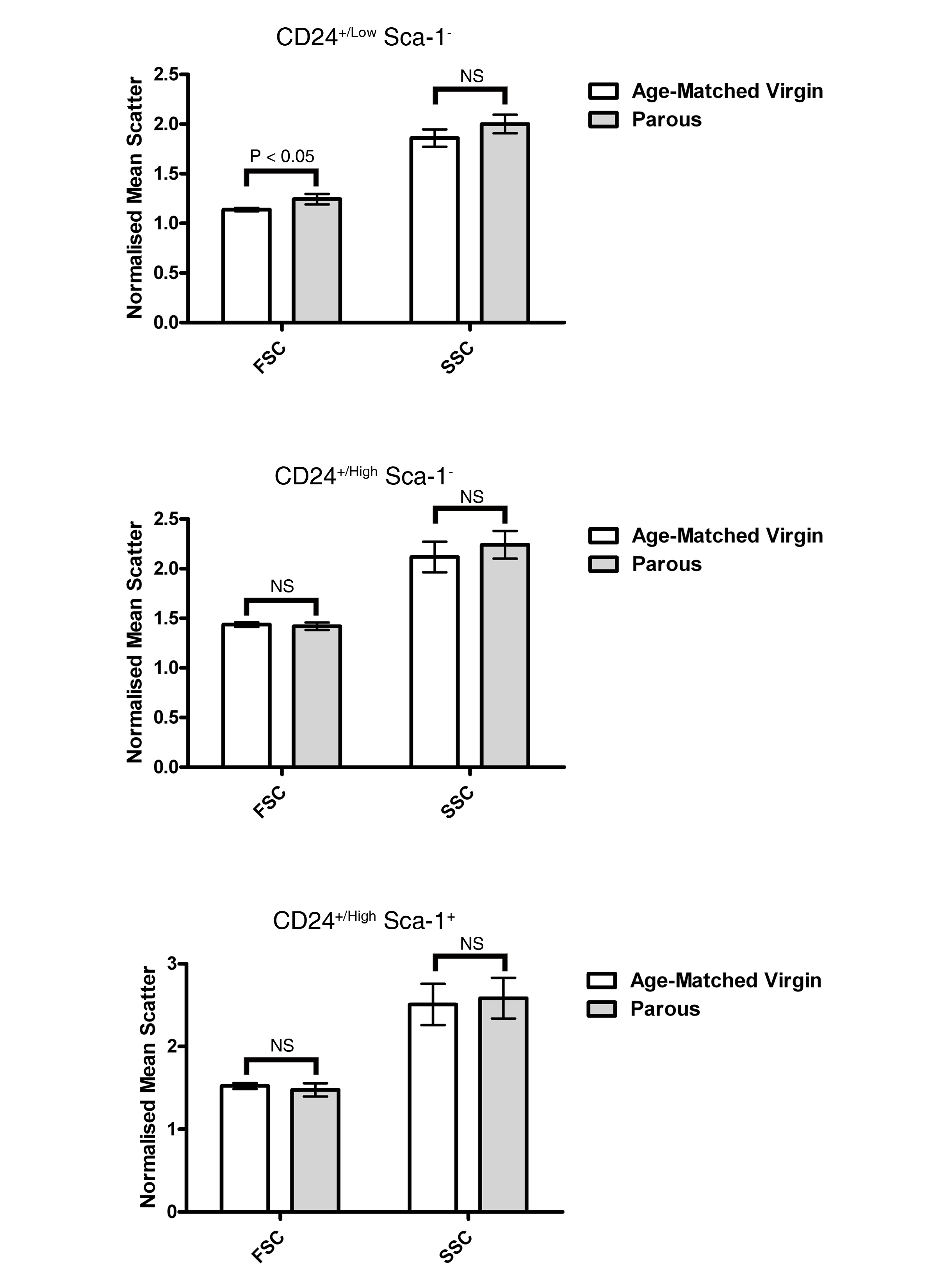

Supplement: Additional file 3 — An Adobe Photoshop TIF file containing an image that shows changes in normalised mean forward scatter/side scatter values of mammary epithelial cell subpopulations between parous and AMV mammary tissue. The figure shows mean forward scatter and side scatter values normalised to lymphocyte forward scatter and side scatter values in AMV and parous tissue for the CD24+/Low Sca-1- (basal; top), CD24+/High Sca-1- (luminal ER-; middle) and CD24+/High Sca-1+ (luminal ER+; bottom) populations (n = 3 independent sorts). [file bcr2245-S3.tiff]
